# Exploring the Development of Existing Sex Education Programmes for People with Intellectual Disabilities: An Intervention Mapping Approach

**DOI:** 10.1111/jar.12017

**Published:** 2012-12-26

**Authors:** Dilana Schaafsma, Joke M T Stoffelen, Gerjo Kok, Leopold M G Curfs

**Affiliations:** * Work and Social Psychology, Maastricht UniversityMaastricht, the Netherlands; † Gouverneur Kremers CentrumMaastricht, The Netherlands; ‡ Clinical Genetics, Maastricht UniversityMaastricht, The Netherlands

**Keywords:** assessment, intellectual disabilities, Intervention Mapping, sex education, sexual health, sexuality

## Abstract

**Background:**

People with intellectual disabilities face barriers that affect their sexual health. Sex education programmes have been developed by professionals working in the field of intellectual disabilities with the aim to overcome these barriers. The aim of this study was to explore the development of these programmes.

**Methods:**

Sex education programmes geared to people with intellectual disabilities were examined in the context of the Intervention Mapping protocol. Data were obtained via interviews with the programme developers.

**Results:**

All programmes lack specific programme outcomes, do not have a theoretical basis, did not involve members of relevant groups in the development process and lack systematic evaluation.

**Conclusions:**

Based on our findings and the literature, we conclude that these programmes are unlikely to be effective. Future programmes should be developed using a more systematic and theory- and evidence-based approach.

## Introduction

In the past two decades, more knowledge has become available on how to develop better health promotion programmes. This influences how we develop sex education programmes nowadays. We now know that providing people only with information will not make them change their behaviour. Behaviour is also influenced by other determinants, like our attitude towards the behaviour, our confidence about performing the behaviour (self-efficacy) and how the behaviour is perceived by others (social norms) (Schaalma *et al*. 2004; Kirby & Laris 2009).

People with intellectual disabilities face challenges in the area of sexuality that might differ from the challenges their non-disabled peers face. For example, people with intellectual disabilities tend to be less informed about sexuality, have fewer sexual experiences, have more negative attitudes towards sexual activities and have more experience with sexual abuse, often as victims, than those without intellectual disabilities (McCabe 1999; Murphy & O'Callaghan 2004; Servais 2006). These problems affect their sexual health and, consequently, their quality of life. Studies have shown that sex education can influence important determinants, like social or behavioural skills (Miltenberger *et al*. 1999; Egemo-Helm *et al*. 2007; Hayashi *et al*. 2011), decision-making skills (Khemka *et al*. 2005) and knowledge (Lindsay *et al*. 1992; McDermott *et al*. 1999) in a positive way.

The aim of our study was to explore the development of existing sex education programmes. Sex education programmes have been developed by professionals who work in the field of intellectual disability to provide direct-care staff and parents a tool for improving the sexual health of people with intellectual disabilities. Little is known regarding how these sex education programmes are developed and how effective they are. Interviews were held with the developers of the programmes. Intervention Mapping (Bartholomew *et al*. 2011) was used as a guideline.

Intervention Mapping provides a systematic framework to the development of theory- and evidence-based programmes (Bartholomew *et al*. 2011). Intervention Mapping describes the process of programme development in six steps: (i) needs assessment, (ii) specifying programme outcomes, (iii) selecting theory- and evidence-based intervention methods and practical applications, (iv) designing and organizing the programme, (v) specifying adoption and implementation plans and (vi) generating an evaluation plan.

In the first step, the needs assessment, the problem for which a programme should be developed, is analysed. In this step, programme developers explore the groups involved or impacted by the problem, the behaviours related to the problem, environmental factors influencing the problem and the relevant psycho-social determinants of the problem. This information can be obtained by doing qualitative and/or quantitative research. A qualitative technique that is being explored for eliciting responses in our particular target population, people with intellectual disabilities, is the Nominal Group technique (Tuffrey-Wijne *et al*. 2007). After the analyses have been carried out, adequate decisions can be made for prioritizing behaviours, expected outcomes, environmental conditions and their determinants for change.

In the second step, programme developers determine the expected outcomes of the programme, which are based on theory, evidence and input from relevant groups, like members of the target population and implementers. When formulating these programme outcomes, it is important to state what the target group needs to learn and do as a result of the intervention. Also in this step, change outcomes are formulated. This change outcomes link the determinants that need to be changed to specific and realistic performance outcomes for the desired behavioural and environmental outcomes.

In the third step, programme developers select theory- and evidence-based methods that can be used to change the psycho-social determinants of the behaviours that were identified in the first step and converted into outcomes in the second step. All conditions for the method must be met in order for it to be effective. The methods subsequently need to be translated into practical applications, which are actual materials and activities that fit the specific target group and the context of the intervention. It is important to involve programme implementers and members of the target group in the selection of the theoretical methods and practical applications.

In the fourth step, all of the components that have been developed are put together to make one coherent programme. Plans are made for pilot-testing and the production of materials. Again, it is imperative that members of the target group are involved in the development and testing of the materials.

In the fifth step, the programme is implemented. This fifth step starts as soon as it is clear who the implementers of the programme will be. It is necessary, in this step, to determine how the programme can be efficiently realized on a larger scale. Involvement of the intended programme implementers and implementation decision-makers (e.g. agents involved in policy-making) is thus crucial in this process, as these people can offer insight regarding not only how programme implementers can be motivated to carry out the intervention but also what kind of support is needed during the implementation process.

The final step of Intervention Mapping entails developing an evaluation plan. This involves assessing whether the measurable outcomes stated in the second step have been met.

The adaptation of the Intervention Mapping protocol, which was used for this study, can be found in Table 1.

**Table 1:** Adaptation of the Intervention Mapping process for the interviews

**Needs assessment**
1. Give a description of the problem or problem behaviour.
2. Which factors influence the problem?
3. Give a description of the target group(s) of the programme
**Programme outcomes**
1. Which behaviours and environmental conditions need to be changed in order to improve quality of life?
2. Were the changes formulated in terms of outcomes? And what are the outcomes?
3. While formulating the outcomes, have changeable factors been taken into account?
**Theory- and evidence-based methods and practical applications**
1. Were members of the target group involved in the development of theoretical methods and practical applications?
2. Which theoretical methods have been used and why?
**Programme development**
1. Were members of the target group consulted about the design of the programme?
2. Were the outcomes incorporated in the programme? And in what way?
3. Were the materials tested before implementation?
**Implementation**
1. Were the programme implementers and implementation decision-makers involved?
2. Were potential barriers identified and taken into account? (e.g. policy, motivation, planning, education level)
**Evaluation**
1. Has the programme been evaluated?
2. Which measurement instruments have been used?

## Materials and Methods

### Selected sex education programmes

The sex education programmes were selected if they were about sex education, targeted people with intellectual disabilities, and were currently in use in the Netherlands. We excluded programmes that only focused on providing sex education in school settings and programmes that focused primarily on preventing sexual abuse. A number of sources were used to retrieve information about existing sex education programmes, namely the Internet, sexologists and other professionals working with people with intellectual disabilities. In total, five sex education programmes were selected for this study (Table 2).

**Table 2:** Overview of the sex education programmes described in this article

Number	Content	Receiver^1^	Giver	Group/Individual	Course available
1	Manual/Workbook Four DVDs Four story books	Mild (adults)	Direct-care staff Parents	Both	No
2	Manual Workbook Stickers	Mild/Moderate (>12 years)	Direct-care staff Parents	Individual	Yes
3	Manual CD	Mild	Direct-care staff Parents	Both	Yes
4	Box Manual Set of photographs Drawings Puzzles Set of icons	Moderate^2^	Direct-care staff Parents	Individual	No
5	Box Manual Sheets with 3D images	Mild	Direct-care staff Parents	Both	Yes

1 Level of intellectual disability (mild: IQ 50-70; moderate: IQ 35-50).

2 People who are supposedly not able to form a relationship.

### Sample

Eleven programme developers of five different sex education programmes were interviewed. Two programme developers of one programme were interviewed separately resulting in a total of six interviews (on average ± 60 min.). The number of people interviewed in a single interview ranged from one to four people and included a psychologist, behavioural experts, a communication expert, sexologists and programme designers. Two interviewers carried out all interviews. The programme developers were approached individually by e-mail, and all agreed to participate in the study.

### Procedure and interview topics

In all interviews, one of the two interviewers functioned as the primary interviewer. Five of six interviews were held in a formal setting, either at the university or at the institution where the participant worked. The other interview was conducted at a participant's residence. The interviews were semi-structured. Topics were derived from Intervention Mapping and were as follows: the needs assessment, the programme outcomes, the theoretical methods and applications, the programme development, the implementation and the evaluation (Table 1). The participants received an overview of the topics prior to the interview so that they could prepare in advance.

### Data processing and analysis

The interviews were recorded with a digital voice recorder and then transcribed. To check for and correct any missing or incorrect information, the completed transcripts were sent to the programme developers for feedback. Once approved, the transcripts were imported into a software program for qualitative data analysis (NVivo 8). A coding scheme derived from the topics discussed during the interviews was employed. A second researcher then validated this coding by topic. Discrepancies between the first and second researcher were resolved through discussion and consensus. Once the topic codes were agreed upon, content was further assigned to categories that were later also validated by the second researcher. Then, the list with scores per category was sent to the programme developers for verification. Of the six interviews, four were returned with feedback. The feedback was subsequently incorporated in the existing list of categories and then compiled to make up the final list of categories.

## Results

Based on the data received from the interviews, we divided the information about the needs assessment into two topics, problem description and determinants, and information about the theoretical methods and practical applications into theories, and theoretical methods and practical applications. Therefore, the results section consists of eight topics: problem description, determinants, programme outcomes, theories, theoretical methods and practical applications, programme development, implementation and evaluation.

### Problem description

During the interviews, we found that programme developers had trouble describing the health-related problems that were the reason for developing the programme. This suggests that identifying the problem and its related factors might not be a significant part of the development process. It was mentioned that sexual abuse was a large issue within the group of people with intellectual disabilities as well as sexual problems and other problem behaviour, such as sexually inappropriate behaviour (Table 3). However, no specific health problems were described by the programme developers.

**Table 3:** Description of problem as stated by programme developers

	1	2	3	4	5^1^
Individual level					
Sexual abuse	x	x	–	–	–
Sexual Problems	–	–	–	x	
Other problem behaviour (e.g. inappropriate behaviour)	–	x	–	x	–
Interpersonal level					
Taboo (in environment)	x	–	–	–	–
Lack of confidence (by staff)	x	–	x	x	–
Organizational level					
Lack of adequate material	x	–	–	x	–
Lack of privacy/room	–	x	x	–	–

1 Numbers refer to the programmes in Table 1.

With respect to environmental factors, it was mentioned that on the interpersonal level there was a lack of confidence in the environment to talk about sexuality and that there is a taboo on talking about sexuality. On the organizational level, lack of adequate material and lack of privacy/room were stated as problems.

### Determinants

When the programme developers were asked to give a description of the problem, they generally gave a description of determinants influencing the problem at the individual level (Table 4). These can be divided into two types of determinants, unchangeable and changeable. Unchangeable determinants were, for example, low IQ level and low social–emotional level. And a changeable determinant was, for example, lack of knowledge concerning topics related to sexuality.

**Table 4:** Description of determinants influencing the problem as stated by the programme developers

	1	2	3	4	5
Individual level					
Low IQ level	x	x	x	x	x
Low social–emotional level	–	x	x	x	x
Autism	–	x	–	x	x
Lack of sexual knowledge	x	x	x	x	x
Lack of social skills		x	x	–	–
Negative attitude (towards their sexuality)	–	x	–	–	x
Vulnerable to sexual abuse	–	x	–	–	–
Interpersonal level					
Dependence client	–	x	–	–	–
Vision (on sexuality)	–	x	x	–	–
Internet (access to pornography)	–	x	–	–	–
Organizational level					
Policy (absence of)	–	–	–	x	x

The programme developers of two programmes mentioned determinants on the interpersonal level, such as dependence of client on caregivers, beliefs about sexuality and Internet access to pornography. The programme developers of two other programmes mentioned a determinant on the organizational level, namely the absence of policy.

### Programme outcomes

The outcomes described by the programme developers all focus on the individual level (Table 5). Knowledge (4), tailoring (4) and empowerment (5) were the outcomes most mentioned. Knowledge stands for increasing the knowledge of people with intellectual disabilities in different areas of sexuality. With tailoring developers meant that each programme had to be adjusted according to the clients' needs and wishes. Empowerment was described as teaching someone to make his or her own decisions. Teaching skills were only mentioned by the programme developers of one programme and so was enjoying sexuality. No outcomes on interpersonal or organizational levels were stated.

**Table 5:** Description of programme outcomes as stated by the programme developers

	1	2	3	4	5
Individual level					
Knowledge	x	x	x	–	x
Teaching skills	–	–	x	–	–
Tailoring	x	–	x	x	x
Empowerment	x	x	x	x	x
Enjoying sexuality	–	–	–	–	x

### Theories

Programme developers were asked to describe which theories were incorporated into the programme. The programme developers of one programme mentioned applying communication models to identify a person's communication level and the programme developers of another programme use the hermeneutic circle as a way to get a complete overview of a persons' history, social–emotional level, cognitive level, etc. No theories on influencing behaviour or psycho-social determinants were pointed out.

### Theoretical methods and practical applications

Programme developers gave descriptions of practical applications they incorporated into the programme, and the underlying theoretical methods of these strategies were separately identified by two researchers. It was not checked if all the conditions, governing effective use of the method, were met. This section presents the theoretical methods that were identified in several categories derived from Bartholomew *et al*. (2011) (Table 6). In the category ‘basic methods at the individual level', tailoring was reported by all the programme developers. Other methods used were persuasive communication (2), participation (2), active learning (2), individualization (2), modelling (1), facilitation (1) and reinforcement (1). In the ‘increase knowledge’ category, images were mentioned by all programme developers. Other methods to increase knowledge were increasing memory and understanding (2), providing cues (2), and discussion (1). In the third category ‘change skills’, guided practice was the most often used method (4). Other methods were goal setting (3), setting graded tasks (2) and planning coping responses (1). In the two remaining categories, ‘changing social influence’ (1) was mentioned as a method to change self-awareness and ‘social comparison’ (1) was mentioned as a method to change social influence.

**Table 6:** Overview of the methods used in the five sex education programmes

	Description	1	2	3	4	5
Basic methods at the individual level						
Tailoring	Components of programme are adjusted to learner's characteristics	x	x	x	x	x
Persuasive Communication	Guide learners towards action using arguments	x	–	x	–	–
Participation	Assure high-level engagement of the participants' group in problem solving, decision making and change activities	x	–	x	–	–
Active learning	Learning from goal-driven and activity-based experience	x	x	–	–	–
Individualization	Provide learner with opportunity to have personal questions answered	–	x	x	–	–
Modelling	Providing an appropriate model being reinforced for desired action	x	–	–	–	–
Facilitation	Creating an environment in which barriers are reduced	–	x	–	–	–
Reinforcement	Linking behaviour to a consequence to increase its rate	–	–	x	–	–
Methods to increase knowledge						
Images	Use of artefacts that have similar appearances to some subject	x	x	x	x	x
Increase memory and understanding	Use of images, metaphors, rehearsing or repeating information in own words, and similar applications	x	x	–	–	x
Providing cues	Assure the same cues to be present at time of learning and the time of retrieval	–	x	–	x	–
Discussion	Informal debate on a topic, guiding learner to activate correct schemes	x	–	–	–	–
Methods for changing attitude						
Self-re-evaluation	Cognitive and affective assessment of one's self-image with and without unhealthy behaviour	x	x	x	–	–
Elaboration	Stimulating learner to add meaning to the information	x	–	–	–	–
Anticipated regret	Focus on feeling after unintended risky behaviour	x	–	–	–	–
Methods for changing skills						
Guided practice	Prompt rehearsal of behaviour, discuss the experience and provide feedback	–	x	x	x	x
Goal setting	Prompt planning towards target behaviour	x	x	–	x	–
Setting graded tasks	Starting with easy task and increasing difficulty until target behaviour is performed	x	–	–	x	–
Planning coping responses	Determining potential barriers and present ways to overcome these	x	–	–	–	–
Methods for changing self-awareness						
Awareness raising	Providing information about consequences and alternatives for problem behaviour	–	x	–	–	–
Methods for changing social influence						
Social comparison	Comparison to non-expert others in order to evaluate oneself	–	–	x	–	–

### Programme development

The programme developers of one programme consulted implementers, in this case direct-care staff, during the development of the programme. When it comes to testing the materials, the programme developers of one programme showed the raw material to clients and direct-care staff in different parts of the country. The programme developers of a second programme have shown visual materials to their clients and enlarged the material after receiving feedback that the size was too small. And the programme developers of a third programme showed their clients drawings and found that photographs were clearer to them than drawings. Furthermore, programme developers indicated that people should be cautious when dealing with a client who has a history of or is currently experiencing sexual abuse.

### Implementation

None of the programme developers mentioned involvement of the implementation decision-makers to plan implementation, while only a few barriers for implementation were indicated. First, programme developers of one programme mentioned that the professional caregivers' lack of confidence and norms could be of influence. Programme developers of a second programme mentioned time as a barrier, meaning it takes time to get familiar with the materials and methods of the programme. Finally, it was mentioned that retrenchment and lack of time given by the institution could be of influence. No clear explanations were given on how to overcome these barriers.

### Evaluation

All interviewees mentioned that there was never an evaluation study conducted of their programme.

## Discussion

The purpose of this study was to assess the development of sex education programmes for people with intellectual disabilities, using existing sex education programmes from the Netherlands as an example. The programme developers who were interviewed all share the same passion to improve the sexual lives of those with intellectual disabilities and were therefore highly motivated to participate in this study. The programmes described in this study are all practice-based. An adaptation of the Intervention Mapping steps was used as a guideline to describe the process and to identify blank spaces and potential for improvement.

Sex education for people with intellectual disabilities may be improved in five areas. The first area is the needs assessment. Description of the problem was usually in terms of determinants instead of focusing on the health problem in terms of behaviour. The determinants that were identified were predominantly on the individual level focusing on the cognitive factor, social–emotional factors and amount of knowledge. Two programmes mentioned determinants on the interpersonal level, and two other programmes mentioned determinants on the organizational level. It is clear that the problems that exist in the area of sexuality of people with intellectual disabilities have to be identified and made more explicit on behavioural level. Only then is it possible to find the related determinants on the individual and environmental levels, which are important for developing an effective programme.

The second area of interest concerns the outcomes stated by the programmes. The ones mentioned most were knowledge, tailoring the programme to the person's need and emancipation. Two of these outcomes, knowledge and tailoring, are represented in methods that were used; however, it was unclear how the outcome of emancipation was reflected in the methods. From the needs assessment and the outcomes stated, it becomes clear that these programmes are only aimed at the target group, people with intellectual disabilities. The question remains whether the outcomes stated by the programme developers are specific and measurable enough. These requirements are important, because a useful evaluation study cannot be conducted if they are not met. Moreover, one should also take environmental outcomes, directed to implementers and implementation decision-makers, into consideration.

The third area concerns the theoretical basis of the programmes. The practical applications that are used are generally chosen based on previous positive experiences and the methods in general lack a theoretical basis, which has a large impact on the effectiveness of the programme because research has shown that non-theory- and evidence-based programmes are not as effective as theory- and evidence-based programmes (Mullen *et al*. 1985; van Empelen *et al*. 2003; Albarracín *et al*. 2005; Bos *et al*. 2008; Peters *et al*. 2009; van Achterberg *et al*. 2010; de Bruin *et al*. 2010). The theoretical models mentioned were mostly applied for identifying a persons' level of IQ, social–emotional level or communication level.

The fourth area concerns the involvement of the target groups, programme implementers and implementation decision-makers. The involvement of these groups during the developmental process was minimal. One programme mentioned involvement of implementers (direct-care staff), but the involvement was minimal; no involvement was mentioned of implementation decision-makers. Also, the involvement of the target group (people with intellectual disabilities) was limited to testing materials in two occasions. This lack of involvement is very concerning. Involvement of the target group is very important for the content and development of materials, and involvement of implementers is very important for recognizing potential barriers for implementation. Finally, implementation decision-makers are very important when it comes to policy; it is next to impossible to implement a sex education programme without good management support (Mendel *et al*. 2008).

The fifth area concerns the evaluation of the programmes. None of the programmes have been evaluated, which is not uncommon (Kok *et al*. 2009). As a consequence, there are no data on the effectiveness of these programmes.

A comparable study that has been conducted by Godin *et al*. (2007), in which they assessed community-based interventions on HIV/STD prevention, found similar results: lack of a proper needs assessment, no specific goals described, lack of theoretical basis and absence of evaluation studies. However, they did not include the involvement of relevant groups in the development process in their assessment tool.

In summary, it is clear that programme developers are very committed and have put much time and effort into making these programmes, but when we look at the development process of these programmes, we can conclude that based on our findings and on the literature the programmes will most likely not be effective. Even more so, due to the lack of measurable outcomes, it will be impossible to do a useful evaluation study (Rossi and Freeman, 1993; pp. 218). Furthermore, we would like to note that even though we know from the literature that different problems exist in the area of sexuality (McCabe 1999; Murphy & O'Callaghan 2004; Servais 2006), we found it remarkable that problems, such as people with intellectual disabilities having fewer sexual experiences, having negative attitudes towards sexual activities and having experiences of sexual abuse, were not as frequently mentioned as was to be expected beforehand.

### Limitations

There are some limitations to this study that should be taken into consideration. The first is that results reflect what is said during the interview by the programme developers and it does not reflect the actual content of the sex education programme. Therefore, it might be possible that they have forgotten to mention important issues due to their lack of knowledge about the Intervention Mapping process. We did try to solve this problem by giving the programme developers an opportunity to provide feedback on two occasions. Furthermore, the practical applications that were mentioned were scored by two researchers and put in different categories of methods. It was not checked if all the conditions for using the method were effectively met.

Finally, it must be taken into account that Intervention Mapping itself has its limitations. It is a protocol and not a method for developing interventions. The quality of the interventions that are developed according to the Intervention Mapping process heavily depends on knowledge that is already out there, and time and money for doing extra research, if that proves to be necessary. Knowledge on the problem behaviour and its determinants is the foundation of a good intervention (Bartholomew *et al*. 2011). When this knowledge is incomplete, it can be expected that this has consequences for the effectiveness of the intervention. The advantage of systematically developing interventions and writing down the process of development is that it increases the likelihood that interventions can be successfully adapted to other settings, knowledge on what is effective can be more easily identified and shared, and aspects of the intervention that turn out not to be effective can be identified and adjusted accordingly.

### Recommendations

For the development of sex education programmes, it is very important to do a proper needs assessment and get a clear idea of the problem and the impact it has on the sexual health of people with intellectual disabilities and identify the psycho-social determinants of the behaviours related to this problem. Usually, these can be found on different ecological levels, such as the individual level, interpersonal and organizational levels (Kok, Gottlieb, *et al*., 2008).

Additionally, the interviews make clear that the focus predominantly lies on the target group, namely people with intellectual disabilities who are receiving sex education. However, other groups should be taken into account as well, like the programme implementers, direct-care staff who give sex education, implementation decision-makers and environmental agents who develop policy.

Furthermore, as mentioned in the introduction, previous research has shown that theory- and evidence-based programmes produce larger effects than non-theory- and evidence-based programmes. It would therefore be useful to develop sex education programmes for people with intellectual disabilities that are theory- and evidence-based instead of only practice-based, such as the current programmes.

Moreover, it is crucial to involve members of the target groups and programme implementers in the different stages of development, because it adds to the effectiveness of the programme and increases the chance of a programme to be successfully implemented. In addition, to understand what parts of the programme are effective and what parts need to be improved, an adequate evaluation plan is necessary. However, in order to conduct a proper evaluation, it is essential to have measurable outcomes in a well-implemented programme (Rossi & Freeman, 1993; pp 218), which are well described (van der Knaap *et al*. 2008). It is therefore preferred to have a systematic and theory- and evidence-based approach when developing a sex education programme.

Finally, assessment of intervention programmes by independent professionals working in the field of intervention development is important to assure the quality of these programmes (Brug *et al*. 2010). This study shows that an adaptation of the Intervention Mapping steps can be a useful tool to asses the development process of sex education programmes.

Summarizing, future research is needed to provide more information on what problems are affecting the sexual health of people with intellectual disabilities, and a more systematical and theory- and evidence-based approach, such as Intervention Mapping, should be applied in the development of future sex education programmes for people with intellectual disabilities.
